# Interpretable Feature Generation in ECG Using a Variational Autoencoder

**DOI:** 10.3389/fgene.2021.638191

**Published:** 2021-04-01

**Authors:** V. V. Kuznetsov, V. A. Moskalenko, D. V. Gribanov, Nikolai Yu. Zolotykh

**Affiliations:** ^1^Institute of Information Technologies, Mathematics, and Mechanics, Lobachevsky State University of Nizhni Novgorod, Nizhni Novgorod, Russia; ^2^Mathematics of Future Technologies Center, Lobachevsky State University of Nizhni Novgorod, Nizhni Novgorod, Russia; ^3^Laboratory of Algorithms and Technologies for Networks Analysis, National Research University Higher School of Economics, Nizhni Novgorod, Russia

**Keywords:** feature extraction, variational autoencoder, ECG, electrocardiography, deep learning, explainable AI

## Abstract

We propose a method for generating an electrocardiogram (ECG) signal for one cardiac cycle using a variational autoencoder. Our goal was to encode the original ECG signal using as few features as possible. Using this method we extracted a vector of new 25 features, which in many cases can be interpreted. The generated ECG has quite natural appearance. The low value of the Maximum Mean Discrepancy metric, 3.83 × 10^−3^, indicates good quality of ECG generation too. The extracted new features will help to improve the quality of automatic diagnostics of cardiovascular diseases. Generating new synthetic ECGs will allow us to solve the issue of the lack of labeled ECG for using them in supervised learning.

## 1. Introduction

All the experience gained by the machine learning community shows that the quality of the decision rule largely depends on what features of samples are used. The better the feature description, the more accurately the problem can be solved. The features are used to require their interpretability, since it means the adequacy of the features to the real-world problem.

The traditional way to build a good feature description was to use an expert knowledge. Specialists in a particular subject area offer various methods for constructing the feature descriptions, which are then tested in solving practical problems. Another approach for constructing a good feature description is automatic feature extraction (also called dimensionality reduction).

There is a lot of methods for automatic feature extraction, such as principal component analysis, independent component analysis, principal graphs and manifolds, kernel methods, autoencoders, embeddings, etc. Among the most powerful and perspective approaches, we mention principal graphs and manifolds (Gorban et al., 2008; Albergante et al., 2020) and methods using deep learning (LeCun et al., 2015; Goodfellow et al., 2016).

Variational autoencoders (VAE) are neural networks which allow you to encode the source information and later, on the basis of the encoded information, to obtain a specific object, and further to generate similar objects but from a random set of coded characteristics (Kingma and Welling, 2013; Rezende et al., 2014; Doersch, 2016). Here we examine this method for the problem of automatic electrocardiogram (ECG) generation.

**Figure 1:**
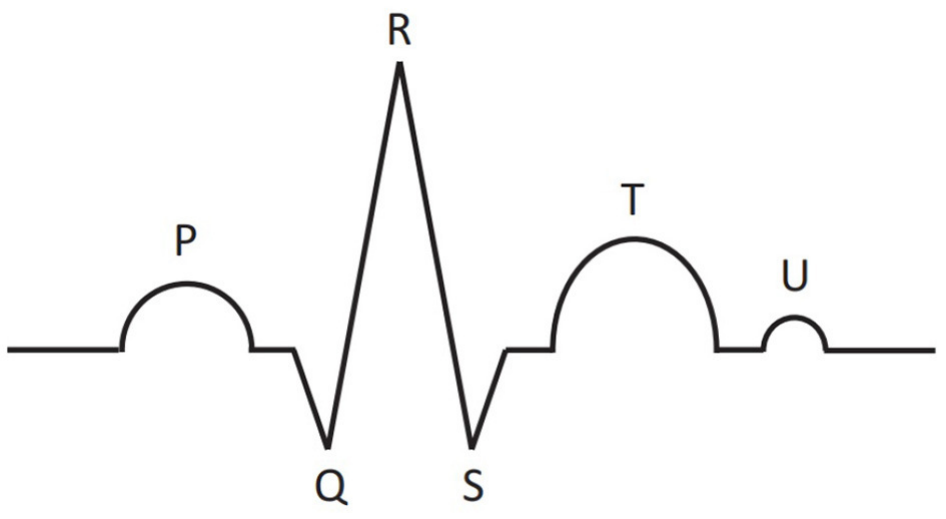
Schematic representation of main parts of the ECG signal for one cardiac cycle: P, T, U waves and QRS complex, consisting of Q, R, and S peaks.

The electrocardiogram is a record of the electrical activity of the heart, obtained with the help of electrodes placed on the human body. Electrocardiography is one of the most important methods in cardiology. Schematic representation of the main part of ECG is shown in Figure 1. One cardiac cycle (the performance of the heart from the beginning of one heartbeat to the beginning of the next) contains P, T, U waves and QRS complex, consisting of Q, R, and S peaks. The size, shape, location of these parts give great diagnostic information about the work of the heart and about the presence/absence of certain diseases.

Recently, machine learning (especially deep learning) methods have been widely used for automatic ECG analysis; see the recent review by Hong et al. (2020). The application tasks include ECG segmentation, disease detection, sleep staging, biometric human identification, denoising, and the others (Hong et al., 2020). A variety of classical and new methods are used. Among them there are discriminant analysis, decision trees, support vector machine, fully-connected and convolutional neural networks, recurrent neural networks, generative adversarial networks, autoencoders, etc. (Schläpfer and Wellens, 2017; Hong et al., 2020).

The most interesting and fruitful directions in applying deep learning methods to ECG analysis are generating synthetic ECGs and automatic extracting new interpretable features. Delaney et al. (2019), Golany and Radinsky (2019), and Zhu et al. (2019) study the problem of ECG generation. The authors of those papers used different variants of generative adversarial networks (GANs) (Goodfellow et al., 2014). The best results concerning the ECG generation were obtained by Delaney et al. (2019). The authors report on the Maximum Mean Discrepancy (MMD) metric equals to 1.05 × 10^−3^.

Our approach in generating ECG is based on VAE. We propose a neural network architectures for an encoder and a decoder for generating synthetic ECGs and extracting new features. The generated synthetic ECGs look quite natural. MMD equals to 3.83 × 10^−3^, which is worse than the value obtained by Delaney et al. (2019) using GAN, but we note that the comparison of these two metric values is not absolutely correct, since the values were obtained on different training sets and for solving similar, but different problems. Qualitatively, the results obtained by the VAE differ from the GAN, but our model is lighter and simpler, and the difference is not colossal. On the other hand, we use VAE, not a regular autoencoder, because VAE will generate signals from a random dataset, which will expand the training sample due to artificially generated ECGs.

The main advantage of our work is the proposal of the method for extracting new features. The goal is to encode data on the signal with the smallest possible number of features. Our experiments show that these features are quite interpretable. This fact allows us to hope that using these features will help to improve the quality of automatic diagnostics of cardiovascular diseases. Generating new synthetic ECGs will allow us to fix the issue of the lack of labeled ECG for using them in supervised learning.

We note that the RR interval is an extremely important parameter of the ECG. Nevertheless, the aim of the study was to generate one cardiac cycle. On the other hand, our approach allows one to generate an ECG and extract features for one cardiac cycle of any duration. Our model is not as large as for the whole signal, and it is convenient to use it in various subtasks related to ECG diagnostics.

Besides VAE, other autoencoders are also used for ECG analysis. In particular, Gyawali et al. (2019) uses f-SAE to capture relevant features from the 12-lead ECG for the downstream task of VT localization. The subject of the work is very different from ours. In our work, we want to use specifically VAE, which can be used for many tasks related to ECG analysis, including for solving our problem.

## 2. Algorithm

### 2.1. Pre-processing

Our original ECG is a 10-s 12-lead signal with a frequency of 500 Hz. Using the segmentation algorithms described by Moskalenko et al. (2019), we determine beginnings and endings of all P and T waves and all the picks R. Then, we do the step forward and backward from the R pick at an equal distance. Thus, we obtain the set of cardiac cycles, each of which of vectors length is 400 (800 ms).

### 2.2. Neural Network Architecture: Encoder

**Figure 2:**
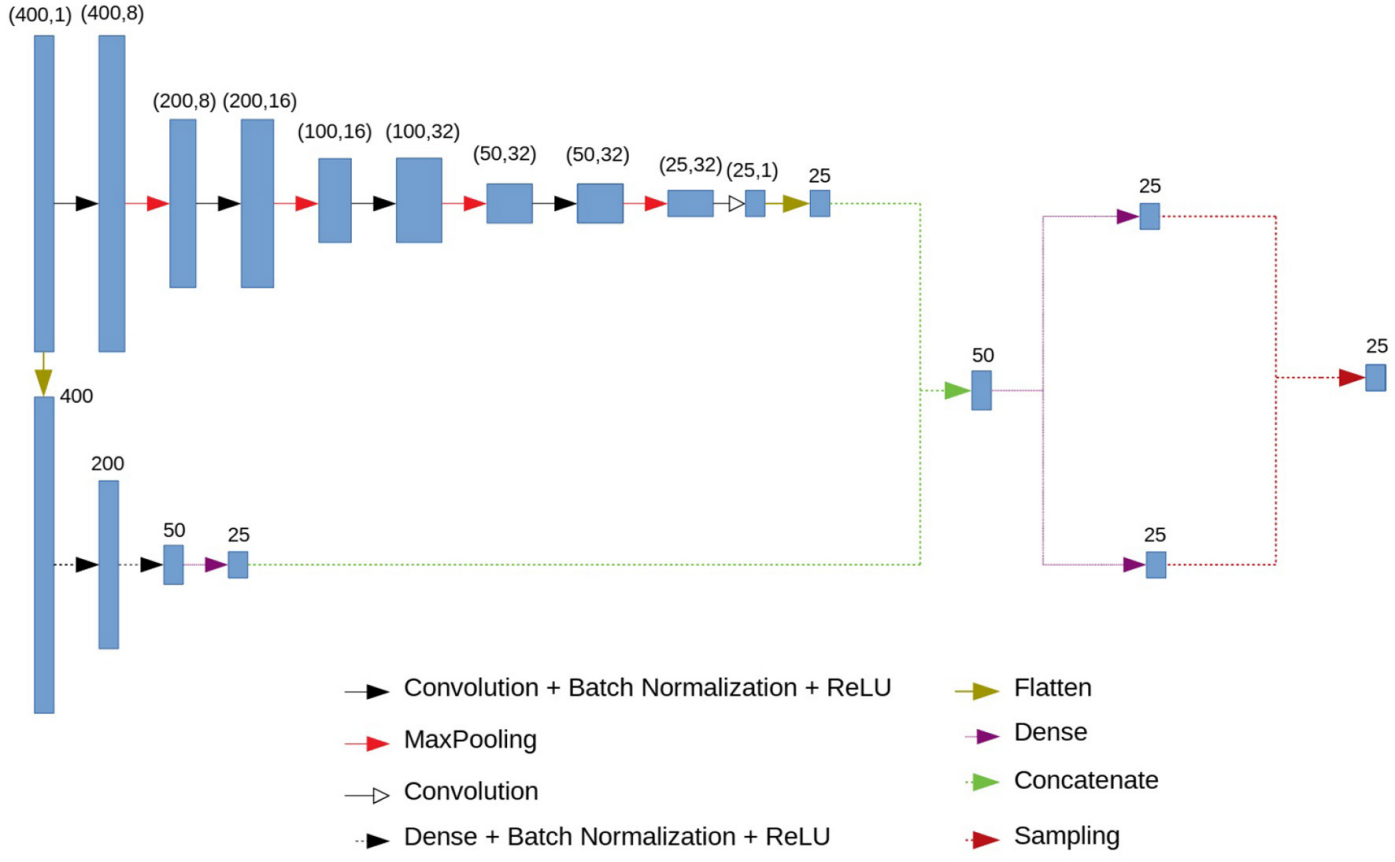
Encoder architecture.

A variational autoencoder (Kingma and Welling, 2013; Doersch, 2016) consists of an encoder and a decoder. We propose the following architecture for them. The encoder consists of a convolutional and a fully connected blocks. The architecture of the encoder is presented in Figure 2. The input vector of length 400 is fed to the input of the encoder. The next step is branching into a fully connected and convolutional chains. This branching occurs immediately in order to simultaneously highlight small local features and features based on the entire signal. Otherwise, using only fully connected blocks, we would get smooth ideal signals, and using only convolutional ones—signals close to a simple set of numbers.

The convolutional chain (at the top of the circuit in Figure 2) consists of four series-connected blocks, each of which consists of a convolution layer, a batch normalization layer, a ReLU activation function and a MaxPooling layer. In addition, we have another convolution layer. At the output of this block we get 25 neurons.

The fully connected chain of the encoder (at the bottom of the circuit in Figure 2) consists of three fully connected (dense) layers, interconnected by a batch normalization and ReLU activation functions. At the output of the last fully connected layer we have 25 neurons.

The outputs of the convolutional and fully connected chains are concatenated, which gives us a vector of length 50. Using two fully connected layers we get two 25-dimensional vectors which are interpreted as a vector of means and a vector of logarithms of variances for 25 normal distributions (or for one 25-dimensional normal distribution with a diagonal covariance matrix). The output of the encoder is a vector of length 25 in which each component is sampled from those normal distributions with specified means and variance.

We will interpret this 25-dimensional vector as a vector of new features sufficient to describe and restore with small error the one cardiac cycle. Note that with fewer features, the results were noticeably worse (the MMD metric was significantly higher). On the other hand, this number of features was enough to restore the signal with sufficient quality.

As the loss function, the Kullback–Leibler distance

(1) $$\begin{array}{l} D_{KL}\left(P∥Q\right)=\int_{X}p\log\frac{p}{q}d\mu \end{array}$$

is used. Due to this fact those 25 new features are of normal distribution. In (1) μ is any measure on *X* for which there exists a function absolutely continuous with respect to μ: $p=\frac{dP}{d\mu }$ and $q=\frac{dQ}{d\mu }$, *P* is the initial distribution, *Q* is the new distribution we have obtained.

### 2.3. Neural Network Architecture: Decoder

**Figure 3:**
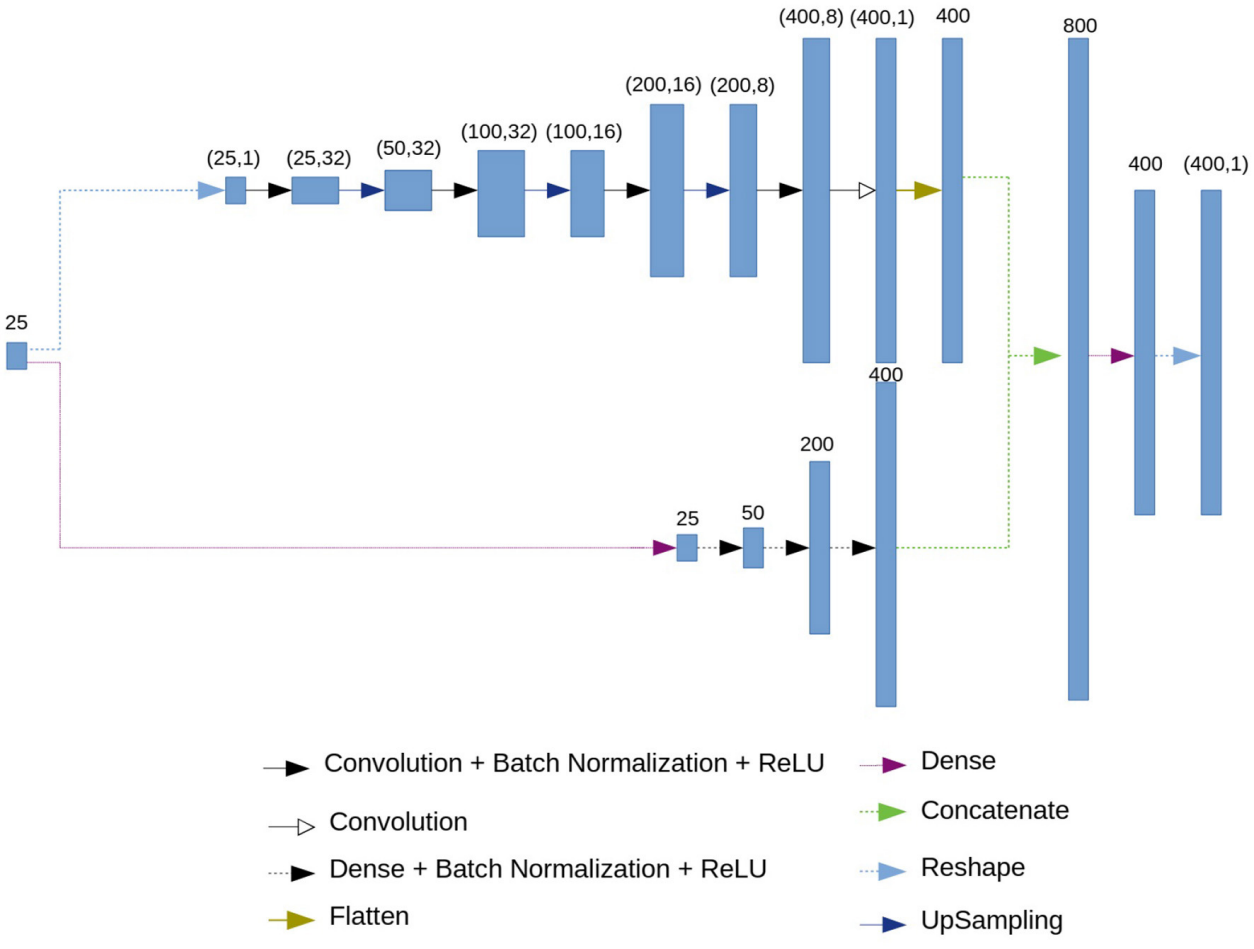
Decoder architecture.

The architecture of the decoder is presented in Figure 3. As an input, the decoder accepts the 25-dimensional vector of features. Then, similarly to the encoder, branching into convolutional and fully connected chains occurs.

The fully connected chain (at the bottom of the circuit in Figure 3) consists of four blocks, each of which contains a fully connected (dense) layer, batch normalization layer and the ReLU activation function.

The convolutional chain (at the top of the circuit in Figure 3) performs a deconvolution. It consists of four blocks which include a convolutional layer, a batch normalization layer, and ReLU activation function, followed by an upsampling layer.

As a result of the convolutional and the fully connected chains, we get 400 neurons from each. Then, we concatenate two results, obtaining 800 neurons. Using a dense layer we get 400 neurons which represent the restored ECG.

As a loss function for the output of the decoder, we use the mean squared error.

The models for the encoder and the decoder can be downloaded from https://github.com/VlaKuz/ecg_cycle_VAE.

## 3. Experimental Results

**Figure 4:**
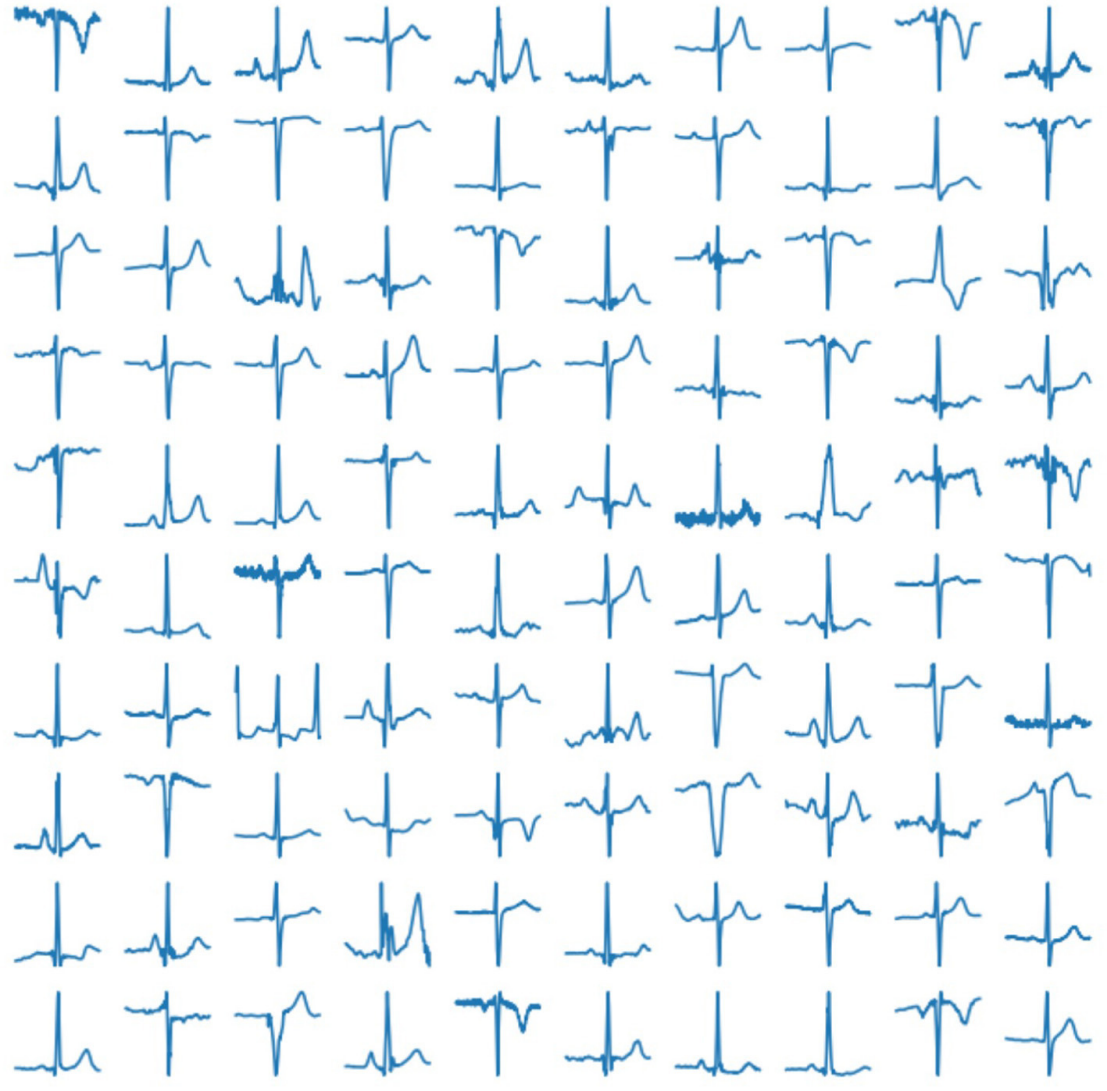
Examples of real cardiac cycles obtained from ECG signals and used in the training of VAE.

In our experiment, we use 2, 033 10-s ECG signals of frequency 500 Hz (Kalyakulina et al., 2019, 2020a,b). We process them according to the principles as described above (see section 2.1) and train our network on the obtained 252, 636 cardiac cycles. Examples of those real human cardiac cycles derived from ECG signals are presented in Figure 4.

To train the model we used 720 epochs of Adaptive Moment Estimation (Adam) algorithm proposed by Kingma and Ba (2014) and implemented in TensorFlow Framework (Abadi et al., 2016). No data augmentation was not performed.

**Figure 5:**
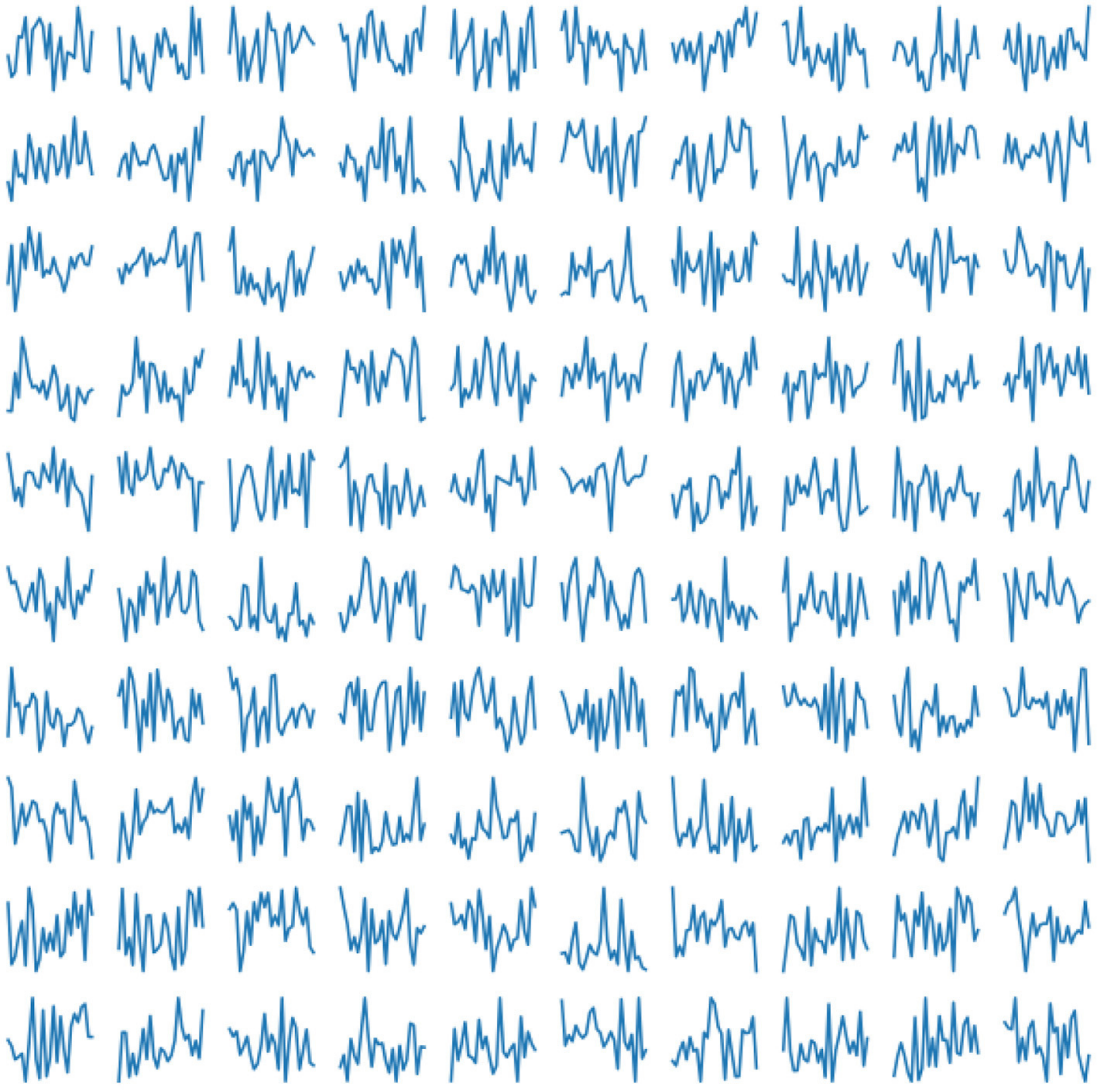
Examples of generated normal distribution features for obtaining a cardio cycle based on them.

The trained network produce 25 features describing the cardiac cycle. The examples are shown in Figure 5.

**Figure 6:**
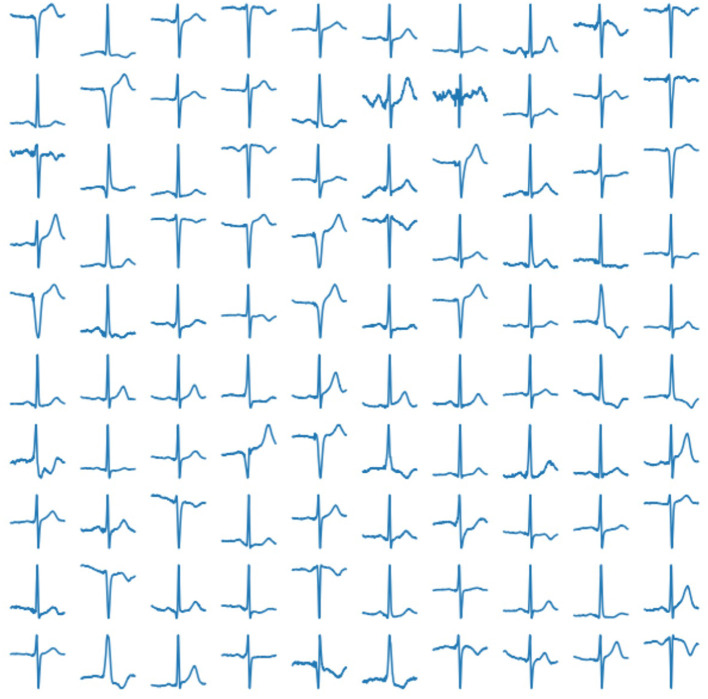
Examples of generated heart cycles based on 25 features.

After having trained the network we may test the decoder by supplying random (generated according to the standard normal distribution) numbers to its input. The examples of the produced results are given in Figure 6. These synthetic generated ECG looks quite natural.

To evaluate our results we calculated the Maximum Mean Discrepancy (MMD) metric (Delaney et al., 2019) on the set of 3,000 generated ECG. The value of MMD is equal to 3.83 × 10^−3^. Keep it in mind that the best value of MMD obtained by Delaney et al. (2019) by GAN is 1.05 × 10^−3^. The value obtained by us is slightly less than the value from Delaney et al. (2019). However, it shouldn't be argued that this metric is a reference. There are no illustrations in Delaney et al. (2019) confirming the correctness of the result. We note that the comparison of these two metric values is not absolutely correct, since these values were obtained on different training sets and for solving similar, but different problems. Unfortunately, the papers (Golany and Radinsky, 2019; Zhu et al., 2019) don't contain (applicable to our problem) values of similar metrics.

**Figure 7:**
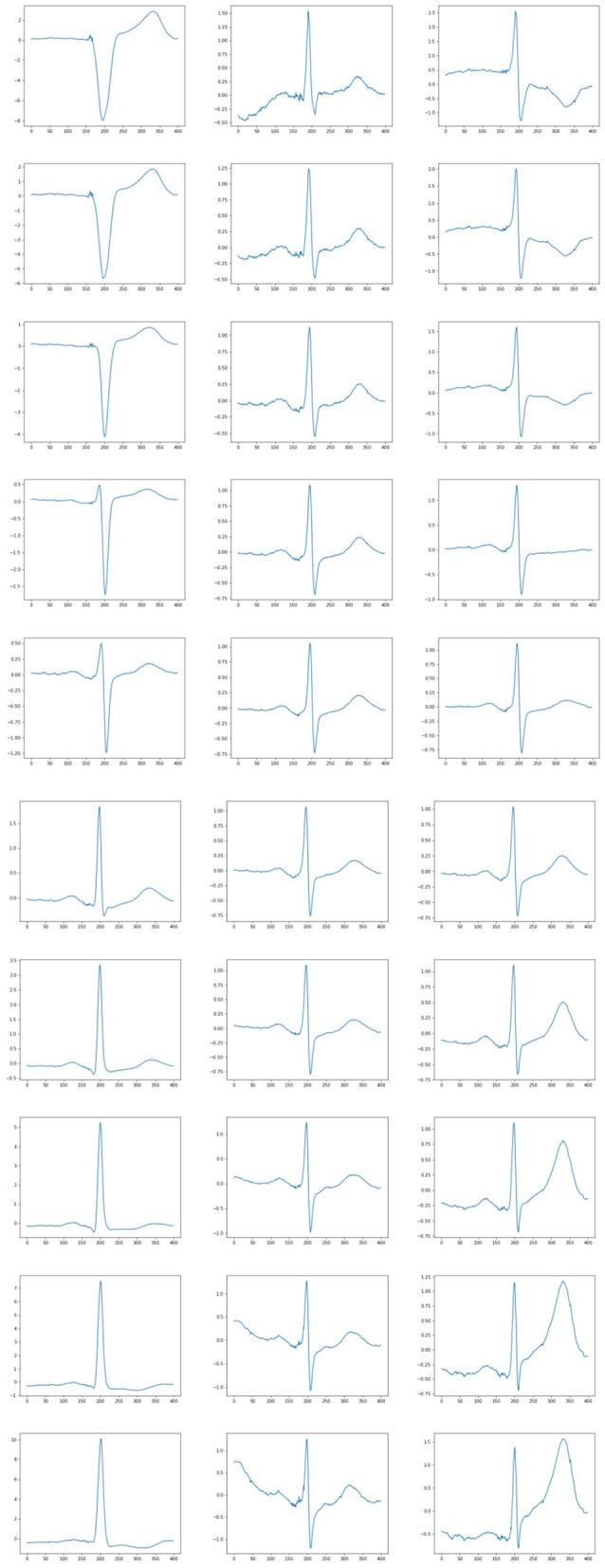
Examples of ECG generated when a parameter is varying. Each column correspond to the set of fixed 24 features and varying other feature (6, 14, and 24 feature, respectively).

Interesting results were obtained when generating ECG with a varying feature. Some generated ECG signals are presented in Figure 7. Twenty-four features were fixed for each test when the remaining feature was changing. It was possible to find a parameter responsible, for example, for the height of the wave T, the depression of the ST wave, etc. Thus, in some cases, the extracted features may be interpreted, which also confirms the high quality of the constructed feature description. So, from the figures it can be seen that when fixing the 6th sign of changes in the behavior of the QRS complex. When the 14th feature changes, the amplitude of the P wave changes, and when the 24th feature changes, the behavior of the T wave changes. Other signs have a similar effect. In all cases, it can be seen that with an increase in the value of the feature, the peak rises up, and with a decrease, it goes down.

**Figure 8:**
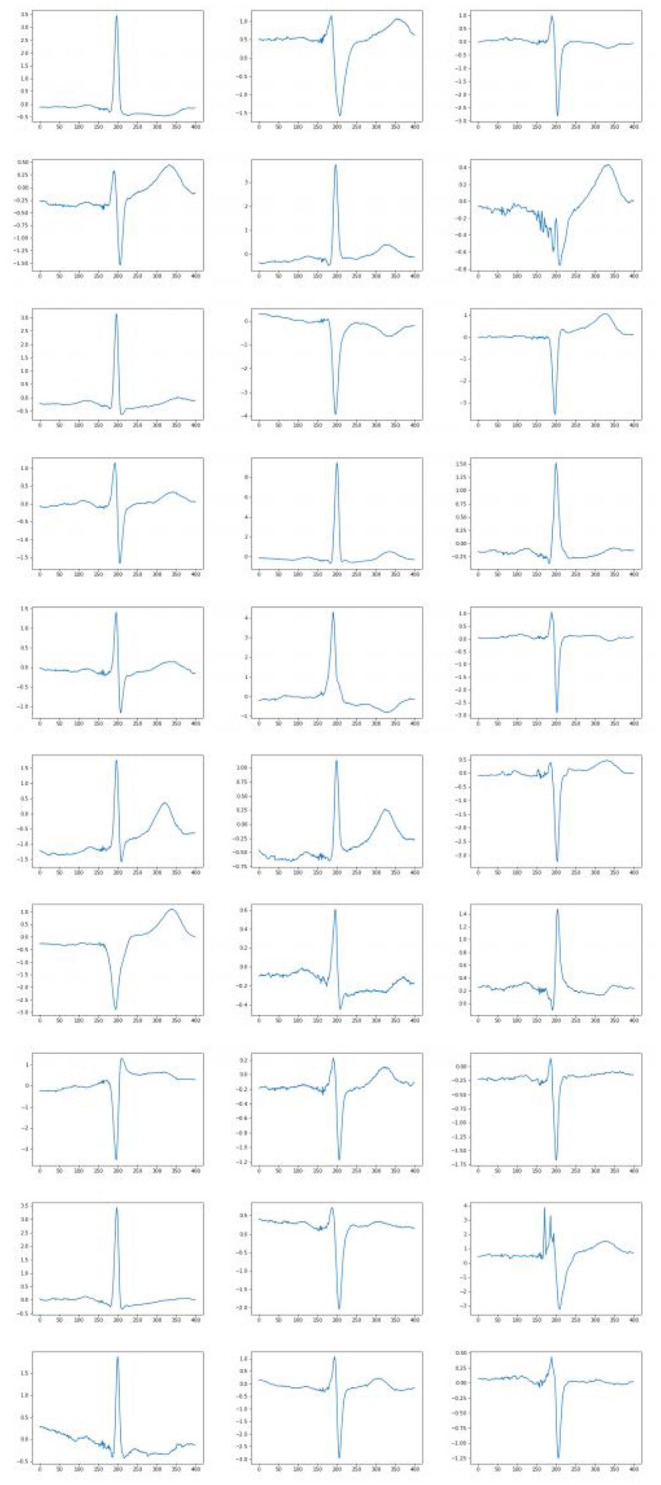
Examples of ECGs generated by a VAE that has been trained in only one lead (I, II, III).

The variational autoencoder models for each lead were also trained. Examples of the results of trained models in the Figure 8. The figure shows the leads I, II, III.

## 4. Conclusions and Further Research

In this paper, we proposed a neural network (variational autoencoder) architecture that is used to generate an ECG corresponding to a single cardiac cycle. Our method generates synthetic ECGs using rather small number (25) of features, with completely natural appearance, which can be used to augment the training sets in supervised learning problems involving ECG. Our method allowed us to extract new features that accurately characterize the ECG. Experiments show that the extracted features are usually amenable to good interpretation.

Our approach has both advantages and disadvantages.

The advantages include relative simplicity, lightness and small size of the system, which makes it very mobile and convenient; the information content of the extracted features by the encoder; the ability to obtain signals from a random distribution of a relatively small number of features; the ability to generate individual signals from a random distribution, as well as generating pathological signals.

The main of the disadvantages is inability to generate a whole ECG signal.

We plan to use our approach to generate the entire ECG, not just one cardiac cycle and, separately, for normal and pathological ECGs cases. We will also use the extracted features to improve the quality of automatic diagnosis of cardiovascular diseases.

## Data Availability Statement

Publicly available datasets were analyzed in this study. This data can be found here: https://physionet.org/content/ludb/1.0.1/.

## Author Contributions

NZ conceived and supervised the study. VK and VM developed the method, performed the experiments and analysis. VK, NZ, and DG wrote the paper. All authors have read and agreed to the published version of the manuscript.

## Conflict of Interest

The authors declare that the research was conducted in the absence of any commercial or financial relationships that could be construed as a potential conflict of interest.
